# Early alpha-lipoic acid therapy protects from degeneration of the inner retinal layers and vision loss in an experimental autoimmune encephalomyelitis-optic neuritis model

**DOI:** 10.1186/s12974-018-1111-y

**Published:** 2018-03-07

**Authors:** Michael Dietrich, Niklas Helling, Alexander Hilla, Annemarie Heskamp, Andrea Issberner, Thomas Hildebrandt, Zippora Kohne, Patrick Küry, Carsten Berndt, Orhan Aktas, Dietmar Fischer, Hans-Peter Hartung, Philipp Albrecht

**Affiliations:** Department of Neurology, Medical Faculty, Heinrich-Heine University Düsseldorf, Moorenstr. 5, 40225 Düsseldorf, Germany

**Keywords:** Lipoic acid, EAE-ON, Optical coherence tomography, Optokinetic response, Multiple sclerosis, Neurodegeneration

## Abstract

**Background:**

In multiple sclerosis (MS), neurodegeneration is the main reason for chronic disability. Alpha-lipoic acid (LA) is a naturally occurring antioxidant which has recently been demonstrated to reduce the rate of brain atrophy in progressive MS. However, it remains uncertain if it is also beneficial in the early, more inflammatory-driven phases. As clinical studies are costly and time consuming, optic neuritis (ON) is often used for investigating neuroprotective or regenerative therapeutics. We aimed to investigate the prospect for success of a clinical ON trial using an experimental autoimmune encephalomyelitis-optic neuritis (EAE-ON) model with visual system readouts adaptable to a clinical ON trial.

**Methods:**

Using an in vitro cell culture model for endogenous oxidative stress, we compared the neuroprotective capacity of racemic LA with the R/S-enantiomers and its reduced form. In vivo, we analyzed retinal neurodegeneration using optical coherence tomography (OCT) and the visual function by optokinetic response (OKR) in MOG_35–55_-induced EAE-ON in C57BL/6J mice. Ganglion cell counts, inflammation, and demyelination were assessed by immunohistological staining of retinae and optic nerves.

**Results:**

All forms of LA provided equal neuroprotective capacities in vitro. In EAE-ON, prophylactic LA therapy attenuated the clinical EAE score and prevented the thinning of the inner retinal layer while therapeutic treatment was not protective on visual outcomes.

**Conclusions:**

A prophylactic LA treatment is necessary to protect from visual loss and retinal thinning in EAE-ON, suggesting that a clinical ON trial starting therapy after the onset of symptoms may not be successful.

**Electronic supplementary material:**

The online version of this article (10.1186/s12974-018-1111-y) contains supplementary material, which is available to authorized users.

## Background

Multiple sclerosis (MS) is an inflammatory autoimmune disorder that involves demyelination, oligodendrocyte death with subsequent axonal damage, and eventually loss of neurons in the central nervous system [1]. In the course of the disease, activated immune cells release mainly nitric oxide (NO) and other reactive oxygen species (ROS), leading to oxidative stress and contributing to the detrimental process of demyelination, axonal damage, and inflammation in both MS [2–5] and its animal model experimental autoimmune encephalomyelitis (EAE) [6, 7]. Especially in active phases of MS and in MS plaques, increased levels of free radicals along with particularly low levels of important antioxidants such as glutathione and vitamin E have been reported [8], suggesting a benefit of antioxidative and neuroprotective substances. However, until now, the vast majority of antioxidative therapeutic trials failed [9, 10], possibly due to the fact that readouts for neurodegeneration were not sensitive enough for short-term changes and not only detrimental aspects of reactive oxygen species (oxidative distress), but also essential signaling functions (oxidative eustress) were affected [9, 11].

Alpha-lipoic acid (LA), a naturally occurring sulfhydryl compound, is found in almost all plants and animals. It has strong antioxidant and anti-inflammatory properties [12]. Some authors have suggested that the (R)-enantiomer of LA is more potent [13] or that its reduced form dihydrolipoic acid (DHLA) is mainly responsible for the antioxidant effects [14]. A previous study has compared the efficacy of the different forms in EAE and demonstrated similar effects on inflammatory and clinical endpoints [15]. A randomized controlled clinical trial evaluating the effect of 1200 mg LA per day for 12 weeks on the antioxidant capacity and serum cytokine profiles revealed an increased total antioxidant capacity [16] and a reduction of pro-inflammatory cytokines INF-γ, ICAM-1, TGF-β, and IL-4 in relapsing-remitting MS [17] while the serum levels of TNF-α, IL-6, and MMP-9 and the clinical expanded disability status scale (EDSS) score were unchanged. In a recent phase II clinical study on secondary progressive MS (SPMS), the annualized percent change of brain volume (PCBV) was significantly reduced in patients receiving LA compared to placebo (− 0.21 ± 0.14 vs − 0.65 ± 0.10) [18].

Large conclusive phase III studies on LA efficacy to prevent clinical progression in MS are lacking. This is possibly owing to the fact that neurodegeneration progresses slowly and neuroprotective trials in MS require long observational periods and large sample sizes to evaluate efficacy on clinical outcomes making them very complicated and costly. Therefore, clinical trial designs with highly sensitive readouts for neurodegeneration and protection allowing shorter observational periods and smaller sample sizes are warranted.

In the past years, optical coherence tomography (OCT) has been established as a non-invasive and powerful tool for the evaluation of neurodegeneration in neurologic disorders [19–23]. This technology has been used in several clinical trials using ON as a model for screening protective and regenerative therapeutic approaches [24]. Furthermore, the high resolution of third-generation spectral-domain (SD)-OCT devices renders in vivo retinal imaging in small rodents possible and is therefore gaining an increasing importance in preclinical neurological research [25–29]. Neuroprotective effects of LA have been investigated in EAE, in the animal model of MS [15, 30–34], and in EAE-ON [35] using histological quantification of axons in the optic nerve. We aimed at comparing the neuroprotective properties of the single LA enantiomers and the reduced form using a model of endogenous oxidative stress in vitro and evaluating the neuroprotective effects of a prophylactic vs therapeutic LA treatment in EAE-ON using in vivo outcomes. The main purpose was to investigate if the previously observed preservation of optic nerve axons in EAE-ON translates to in vivo readouts of structure and function, namely OCT and optokinetic response (OKR), which can be applied in a phase II clinical trial on optic neuritis in patients.

## Methods

### Cell culture and glutamate toxicity assay

The HT22 cell line was cultured at 37 °C in a 5% CO2 atmosphere and Dulbecco’s modified Eagle medium (DMEM) high glucose (ThermoFisher Scientific, GIBCO Life Technologies), containing 5% fetal calf serum (Hyclone), 100 U/mL penicillin, and 100 μg/mL streptomycin (GIBCO Life Technologies). For the cell viability assays, 5 × 10^3^ HT22 cells were seeded in a 96-well plate and pre-incubated with either LA or DHLA (Sigma-Aldrich) for 9, 6, and 2 days or 1 day (d-9; d-6; d-2, − 24 h), treated at the same time (0 h), or hours (+ 3 h or + 6 h) after l-glutamate (Sigma-Aldrich) addition. Another 24 h later, cell viability was assessed using the CellTiter-Blue (Promega) assay as previously described [36].

### Mice and induction of EAE

Female, 6-week-old C57BL/6J mice were purchased from Janvier Labs (Le Genest-Saint-Isle, France). Mice were immunized with 200 μg of myelin oligodendrocyte glycoprotein fragment 35–55 (MOG_35–55_)_,_ purchased from BIOTREND emulsified in 200 μl of complete Freund’s adjuvant (CFA), supplemented with 800 μg of heat-killed *Mt.*, H37Ra, both purchased from BD Difco (injected subcutaneous, distributed over four spots on the hind and front flank) and additional intraperitoneal injections of 200 ng of pertussis toxin (PTX) from Sigma-Aldrich on days 0 and 2 after immunization. The sham control group (sham-EAE) also received PTX and CFA, but no MOG_35–55_ peptide. LA stock solution was prepared at 500 μM in dimethylsulfoxid (DMSO) and stored at − 80 °C until use. Treatment started either 1 week before (d-7), at the day of (d0), or 14 days after (d14) induction of EAE by adding LA stock solution (verum) or DMSO alone (vehicle) to the drinking water. Drinking water was replaced twice a week, uptake was measured daily, and the LA concentration was adjusted to a daily treatment dose of 100 mg/kg bodyweight (BW) per day. The clinical EAE score was graded daily according to the following criteria: (0) no disease, (0.5) mild tail paresis, (1) obvious tail paresis or plegia, (1.5) tail plegia and no righting reflex, (2) mild signs of hind limb paresis with clumsy gait, (2.5) obvious signs of hind limb paresis, (3) hind limb plegia; drags one hind limb behind, (3.5) hind limb plegia; drags both hind limbs behind (4) mild signs of quadriparesis (4.5) quadriplegia, and (5) death or moribund.

All animal procedures were performed in compliance with the experimental guidelines approved by the regional authorities (State Agency for Nature, Environment and Consumer Protection; AZ 84-02.4.2014.A059) and conform to the Association for Research in Vision and Ophthalmology (ARVO) Statement for the Use of Animals in Ophthalmic and Vision Research.

### Optical coherence tomography

We report the OCT methodology in line with the APOSTEL recommendations [37], and an APOSTEL checklist is provided as supplementary material (see Additional file 1). The OCT measurements were performed with a Spectralis™ HRA+OCT device (Heidelberg Engineering, Germany) under ambient light conditions. The mice were positioned in a custom OCT holder described elsewhere [38] and anesthetized with isofluran vaporized at concentrations of 2.5% (2 L/min O_2_). Their pupils were dilated with 2.5% phenylephrine-0.5% tropicamide ophthalmic solution (pharmacy of the University Hospital Düsseldorf). For imaging of the mouse retina, we used a custom contact lens and Visc-Ophtal eye gel (Dr. Winzer Pharma) during the examination to keep the eyes moist and to ensure a constant and homogenous refraction. A 25-diopter adaptor lens was placed on the objective lens of the OCT device to adapt the focus to the mouse eye and retina. The OCT imaging was carried out with the software integrated TruTrack™ eye tracking to diminish breathing artifacts and to achieve consistent ocular orientations.

We performed volume scans (25 × 25°) to analyze the thickness of the retinal layers. All scans were acquired with an initial focus distance of 38 diopters followed by manual correction. Each volume scan consisted of 25 B-Scans recorded in high-resolution mode at 50 automatic real time (ART, rasterized from 50 averaged A-Scans). We used automated segmentation by the Heidelberg Eye Explorer™ software version 1.9.10.0 followed by manual correction of a blinded investigator. The thickness measurements were derived from the circular 1, 2, and 3 mm early treatment of diabetic retinopathy study (ETDRS) grid centered on the optic disc, excluding the central part. We used the high-resolution mode; all scans had a quality of at least 20 dB. We calculated the thickness of the inner retinal layers (IRL), consisting of the retinal nerve fiber layer (RNFL), ganglion cell layer (GCL), and inner plexiform layer (IPL) by averaging each sector of the grid, excluding the center which corresponded to the optic nerve head.

### Optokinetic response for visual function analysis

The optokinetic response analysis was carried out with a testing chamber and the OptoMotry™ software from CerebralMechanics™, Lethbride, Canada [39]. The mice were positioned on a platform in a box containing four screens displaying a moving grid creating a virtual cylinder with varying frequencies. The mice were monitored from above by a camera, and the head movements (tracking) were evaluated by an investigator blinded on the experimental groups. As a measure for visual acuity, we used the threshold of the highest spatial frequency at which the 100% contrast moving grid was still tracked by the mice. Clockwise tracking represented the left and counterclockwise the right eye. A more detailed description of the device and methodology is given elsewhere [40].

### Tissue sampling and histological analysis

After 120 days of EAE, mice were sacrificed with an overdose of isofluran (Piramal Critical Care) and cardiac perfusion was performed with cold phosphate-buffered saline (PBS). Brains were extracted, washed in PBS, and frozen; optic nerves and retinae were isolated. Optic nerves were fixated in 4% paraformaldehyde (PFA) over night and dehydrated in sucrose solutions with increasing concentrations. After embedding in O.C.T. compound (Sakura™ Finetek), longitudinal sections of 5 μm were cut for immunohistological analysis. To examine CD3^+^ lymphocyte and microglial infiltration and activation, as well as the myelin status of the optic nerves, slices were incubated with CD3- (1:400, Dako), Iba1- (1:500, Wako chemicals), and myelin basic protein (MBP)- (1:500, Millipore) antibodies, respectively. Cy3 anti-rat and Cy5 anti-rabbit (1:500, Millipore) were used as secondary antibodies. For rating the immune cell infiltration in optic nerves, hematoxylin and eosin (HE) staining was performed. Retinae were fixated in 4% PFA for 30 min and stained with βIII-tubulin antibody (1:1000, Biolegend) and secondary antibody donkey anti-mouse IgG conjugated to Alexa Fluor 488 (1:1000, Invitrogen) for ganglion cell counting.

Microglial infiltration and activation were quantified by fluorescence intensity measurement of the Iba1 staining. The HE and CD3 staining results were rated by an investigator blinded to the experimental groups by a score [41] 0, no infiltration; 1, mild cellular infiltration; 2, moderate infiltration; 3, severe infiltration; and 4, massive infiltration.

The pathologic findings of the MBP staining were graded from 0 to 3 as previously described [42] by a blinded investigator: 0, no demyelination; 1, rare foci; 2, a few areas of demyelination; 3, large/confluent areas of demyelination.

Fluorescence-stained longitudinal optic nerve sections were acquired with a Leica HyD detector attached to a Leica DMi8 confocal microscope (× 63 objective lens magnification) and HE images with a camera (Olympus Color View III) attached to a Olympus BX51 microscope (× 20 objective lens magnification). At least four sections of the optic nerve, exclusively of the right eye of each mouse, were analyzed per staining. The entire longitudinal section of each optic nerve was included for rating and intensity measurement.

### Detection of carbonylated proteins

Cortical brain samples were homogenized with a micro pestle on ice and re-suspended in NP-40 lysis buffer. The homogenate was incubated on ice for 30 min and vortexed every 10 min. Ice cold lysates were then sonicated three times at 10% power using a Bandelin Sonoplus UW2070 sonifier and cleared by centrifugation (4 °C, 12.000*g*, 15 min). Total protein carbonylation level was determined using the OxyBlot Protein Oxidation Detection Kit (Millipore) according to the manufacturer’s protocol; the secondary antibody was replaced by the IRDye™ 680RD-conjugated goat-anti-rabbit (LI-COR Bioscience). The total fluorescence intensities were measured using a Li-COR Odyssey Clx Infrared Imaging System and normalized to β-actin (1:5000, Sigma).

### Quantification of total glutathione

For estimation of reduced glutathione (GSH), 3 × 10^5^ of HT22 cells were seeded in a 60-mm dish with 25 μM LA or vehicle DMSO treatment. After 24 h, glutamate was added at the indicated concentrations for 8 h and cells were harvested with lysis buffer. For measurement of GSH levels in tissue, frozen mouse cortices were homogenized with a micro pestle in PBS/EDTA buffer, sonicated, and transferred to lysis buffer. Tissue and cell samples were further processed and measured enzymatically as described previously [36] with a bicinchoninic acid assay for normalization against whole protein amount.

### Statistics

Statistical analysis was performed using Microsoft Excel and Prism 5.0 (Graphpad). Data of the glutamate toxicity assays were fitted by sigmoid curves using the least squares method to estimate EC_50_. A two-tailed analysis of variance (ANOVA) with Dunnett’s post hoc test was used to compare the area under the curve for the glutamate toxicity assays, OCT courses and cumulative EAE scores. Group means were compared by one-way ANOVA with Dunnett’s post hoc test using the means of both eyes of each animal for in vivo data and one eye per animal for the different histological investigations. Spearman correlations were performed to analyze the association between IRL thickness, OKR and clinical scores.

## Results

### LA and its reduced form protect from oxidative glutamate toxicity

To identify the effective concentrations and compare if both LA enantiomers and the reduced form DHLA are equally bioactive, we investigated their neuroprotective capacities in oxidative glutamate toxicity, an in vitro model of endogenous oxidative stress using the HT22 hippocampal mouse cell line. In this model, high concentrations of extracellular glutamate block the glutamate-cystine antiporter system (xc^−^), resulting in GSH depletion and oxidative damage due to a lack of cellular cysteine.

**Fig. 1:**
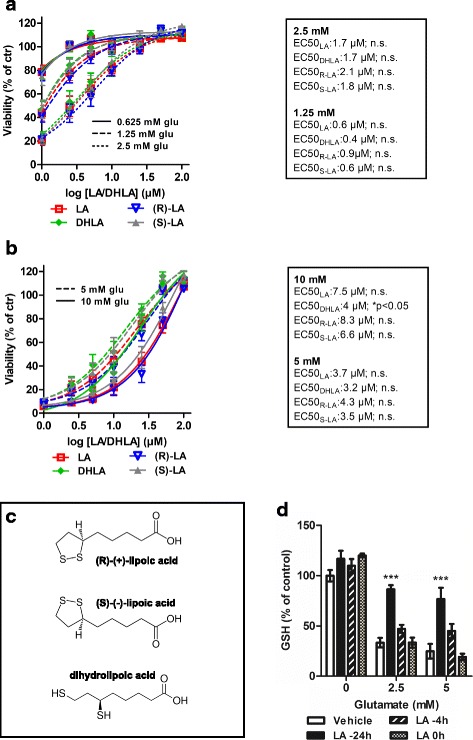
Alpha-lipoic acid as well as its enantiomers and DHLA show protective effects in a model of oxidative glutamate toxicity. Five thousand HT22 cells were seeded into 96-well plates and treated with either vehicle or different concentrations of LA, DHLA, (S)-LA, or (R)-LA, followed by glutamate 24 h later. Graphs represent curve fits ± SEM for 0.625, 1.25, and 2.5 mM (**a**) and 5 or 10 mM (**b**) glutamate (glu) of five independent experiments, each performed in triplicates with corresponding EC_50_ values. Significant differences between LA and DHLA, (R)-LA, or (S)-LA treatments are indicated by asterisks (*p < 0.05, area under the curve compared by ANOVA with Dunnett’s post hoc test). Structural formulas of the used compounds (**c**). For GSH, cells were treated with 25 μM LA for indicated time points and glutamate for additional 8 h before harvesting for protein extraction and enzymatic assay (**d**) (***p < 0.001, compared by two-way ANOVA with Dunnett’s post hoc test to vehicle treatment)

LA or DHLA provided neuroprotective properties when pre-incubated 24 h before glutamate exposure. We observed no difference between the natural occurring (R)-(+)- and the (S)-(−)-enantiomers and similar EC_50_ values (Fig. 1a, b). At 10 mM glutamate, the EC_50_ value of DHLA was significantly improved compared to LA, suggesting that the reduced form has superior antioxidant capacities. The structural differences between the compounds are shown in Fig. 1c. Total intracellular GSH was measured 8 h after glutamate addition. An increase of the GSH levels in LA-treated cells was detected after a pretreatment of 24 h (Fig. 1d).

**Fig. 2:**
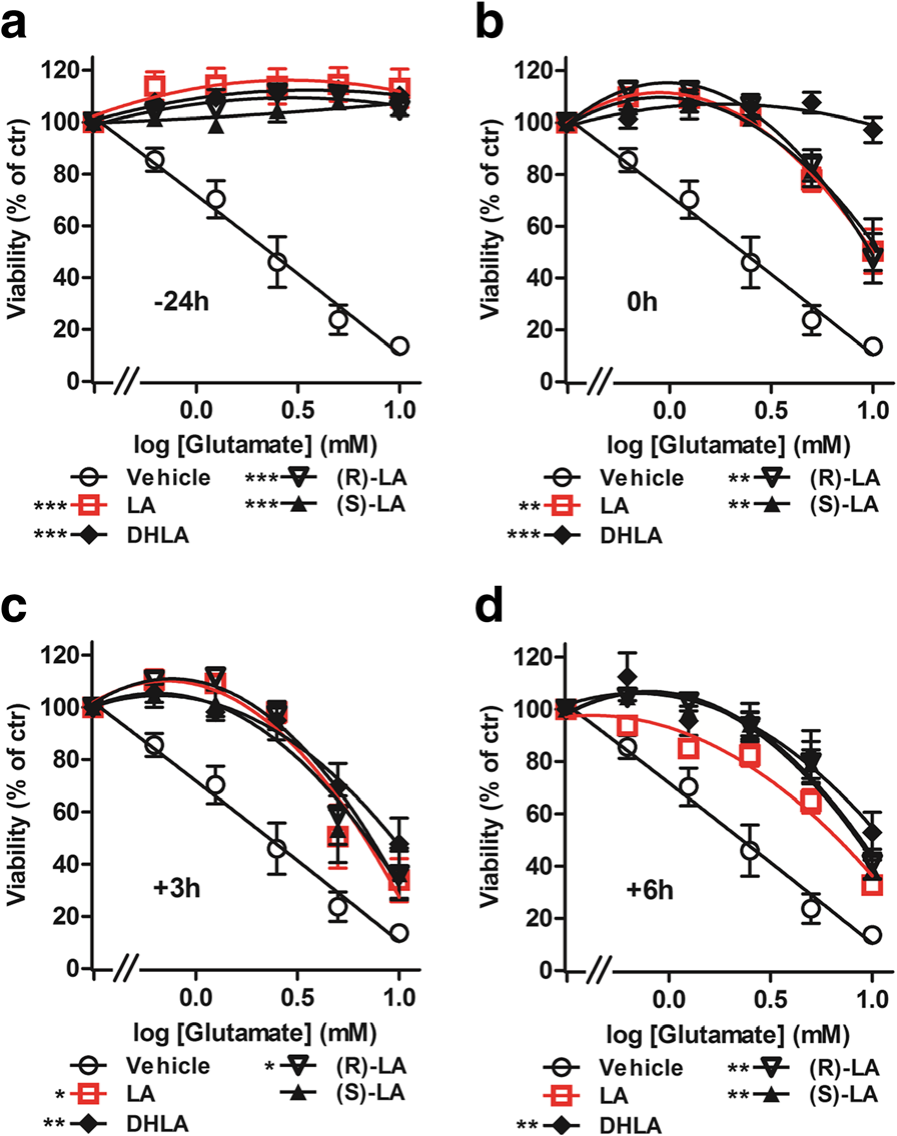
Time course experiments reveal that DHLA acts faster than the precursor LA. Five thousand HT22 cells were seeded into 96-well plates and treated with either vehicle or 25 μM of LA, DHLA, (S)-LA, or (R)-LA starting 24 h before (**a**), at the same time (**b**), 3 h (**c**), or 6 h (**d**) after glutamate addition. Graphs represent curve fits ± SEM of four independent experiments, each performed in triplicates. Significant differences between vehicle and substance treatment are indicated by asterisks (*p < 0.05; **p < 0.01; ***p < 0.001, area under the curve compared by ANOVA with Dunnett’s post hoc test)

To examine the dynamics of these protective effects, several time course experiments were performed. An incubation with LA and its enantiomers 24 h before glutamate treatment led to a saturated protective effect while later treatment resulted in decreased cell survival (Fig. 2). Earlier LA treatment (days 9, 6, or 2), did not result in increased protection for the (R)-(+)- and (S)-(−)- enantiomers or the racemic mixture (see Additional file 2). The protective effect of DHLA was already saturated when it was added at the same time as the glutamate addition, providing further evidence that antioxidant effects are mediated by the reduced form. However, all forms revealed the same protective capacity after a 24-h pretreatment. Our experiments demonstrated a few hour difference only between LA and DHLA. We therefore decided to carry out all following in vivo experiments with the racemic mixture of LA.

### LA reduces the disability score and retinal degeneration in an EAE model

**Fig. 3:**
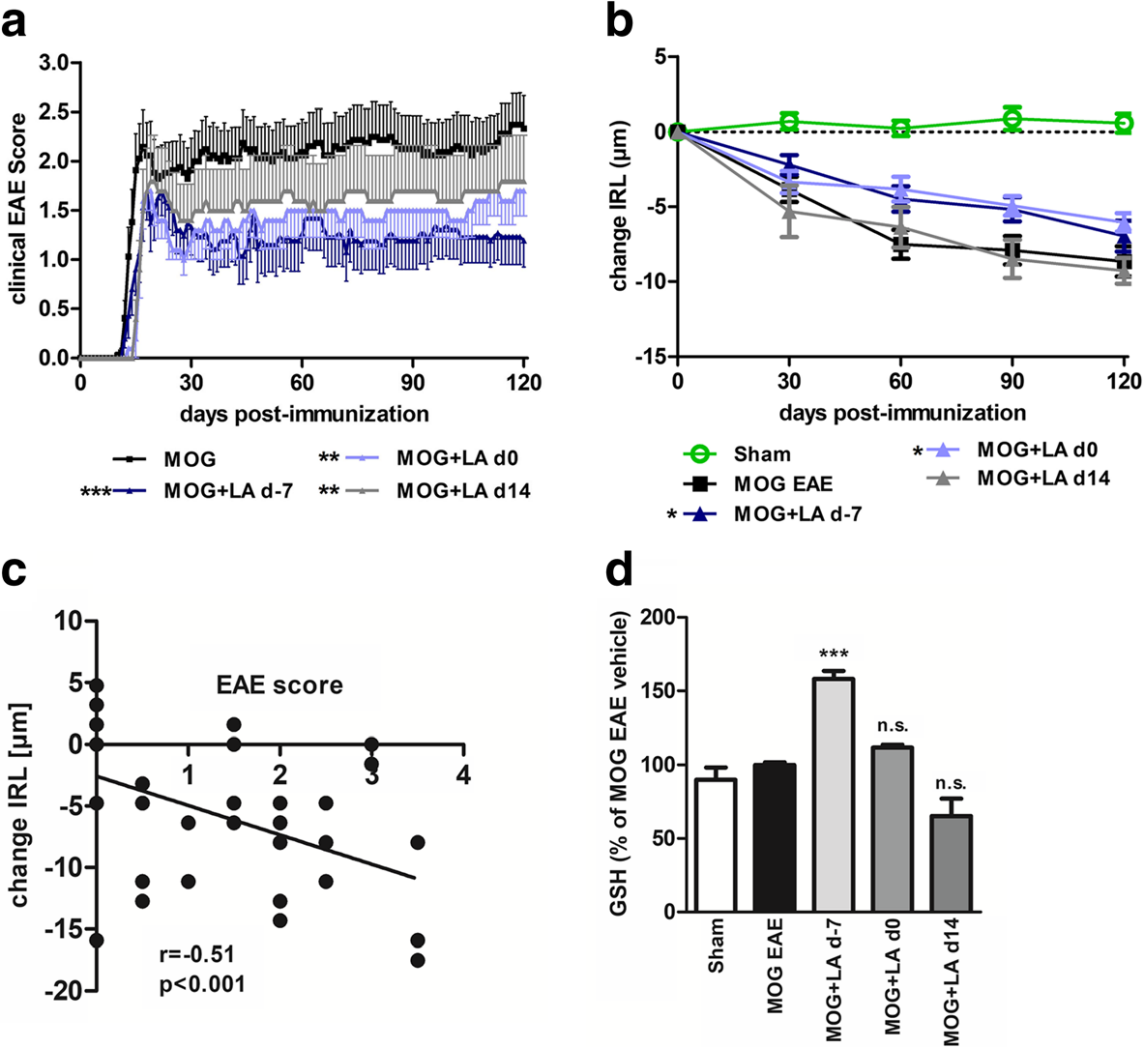
The clinical course of MOG-induced EAE in C57BL/6J was attenuated, and the degeneration of the inner retinal layers was less pronounced in LA-treated mice. Prophylactic LA treatment acted beneficial on clinical EAE scores while later therapy had less pronounced effects (**a**). Thinning of the inner retinal layers was reduced during the EAE only when LA was administered prophylactically at the day of immunization or 7 days before while treatment at the onset of disease at day 14 had no effect (**b**). The correlation between the IRL thickness change and the EAE score at day 120 (**c**). GSH levels in the mouse cortices were significantly reduced after prophylactic LA treatment (**d**). Mice were scored daily, and OCT measurements were performed once a month over 120 days. GSH analysis was performed 120 days after EAE immunization. The time courses and bar graph present the pooled results of three independent EAE experiments with at least four mice per group (*p < 0.05; **p < 0.01; ***p < 0.001, area under the curve compared by ANOVA with Dunnett’s post hoc test for time courses. Associations were calculated with Spearman correlation; each point represents an eye, some data points overlap. ***p < 0.001; n.s. = not significant, by ANOVA with Dunnett’s post hoc test compared to MOG-untreated mice for bar graphs)

In order to test the potential of LA to protect from acute inflammatory relapses like optic neuritis, we investigated retinal neurodegeneration in MOG_33–55_ peptide-induced EAE-ON in C57BL/6J mice. In this model, the rate of optic neuritis was very high with approximately 92% of nerves showing infiltrates in HE-stained longitudinal histological sections. Prophylactic treatment (d-7) with 100 mg/kg BW LA per day reduced the clinical score over a period of 120 days compared to vehicle (DMSO)-treated control mice (*p* < 0.001, one-way ANOVA analysis). A later initiation of LA therapy starting at days 0 and 14 after immunization led to a higher first peak of disease around day 14 and to subsequent higher clinical scores compared to mice treated with LA starting 7 days before MOG injection (*p* < 0.01, one-way ANOVA analysis) (Fig. 3a). Untreated sham control mice showed a nearly constant IRL thickness with minor growth over a period of 120 days, while MOG peptide-immunized animals presented a prominent loss of IRL thickness until day 60 and then a slow and steady decrease continuing until day 120 when they were sacrificed. Prophylactic LA treatment starting 7 days before and at the day of immunization resulted in an attenuated degeneration reflecting the course of the clinical EAE score. Therapeutic LA therapy starting at day 14 at the peak of the disease failed to preserve the inner retinal layers from degeneration (Fig. 3b). The clinical EAE score significantly correlated with the results of the OCT measurements (Fig. 3c) (*r* = − 0.51, *p* < 0.001, Spearman). Prophylactic LA treatment increased GSH levels in the mouse cortex, while later treatment had no effect on the GSH concentration (Fig. 3d). However, the carbonylation of the proteins in the brain was not changed after LA treatment compared to untreated MOG control 120 days after EAE immunization (see Additional file 3).

### Visual function and correlation of OKR and OCT readouts with the clinical EAE score

**Fig. 4:**
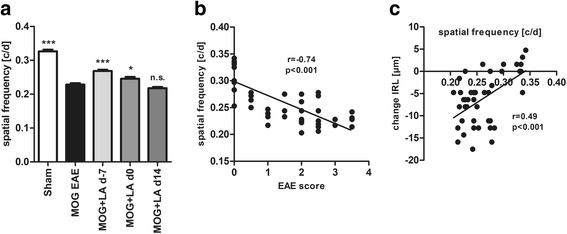
Prophylactic LA treatment improved visual acuity of C57BL/6J mice compared to untreated mice with MOG EAE. OKR measurement was carried out 120 days after MOG immunization as described above (**a**). The bar graph represents the pooled mean ± standard deviation of at least three separate EAE experiments each with at least four animals per group (***p < 0.001, *p < 0.05, n.s. = not significant, by ANOVA with Dunnett’s post hoc test compared to MOG-untreated mice). We found a correlation between clinical EAE score and the spatial frequency threshold from OKR measurements at day 120 (**b**) as well as the OKR values and the thickness change of the inner retinal layers at day 120 (**c**). Associations were calculated with Spearman correlation; each point represents an eye, some data points overlap. Results represent data from three pooled experiments each with at least three mice per group

To analyze the visual function, we assessed the optokinetic response of the mice using the spatial frequency OKR threshold as a surrogate for visual acuity. The spatial frequency threshold was significantly reduced in EAE mice with values of 0.23 cycles per degree (c/d) compared to 0.33 c/d for sham-EAE control mice. A prophylactic LA treatment starting 7 days before or at the day of immunization (d0) reduced visual loss with spatial frequency values of 0.27 and 0.25 c/d, respectively, compared to 0.23 in vehicle-treated mice (*p* < 0.001, one-way ANOVA analysis). Therapeutic treatment starting at the first peak of disease (d14) had no beneficial effect on vision (Fig. 4a). These OKR values correlated significantly with the clinical EAE scores (Fig. 4b) (*r* = − 0.74, *p* < 0.001, Spearman) and inversely with the thinning of the inner retinal layers (Fig. 4c) (*r* = 0.49, *p* < 0.001, Spearman).

### Retinal ganglion cells are preserved after LA treatment

**Fig. 5:**
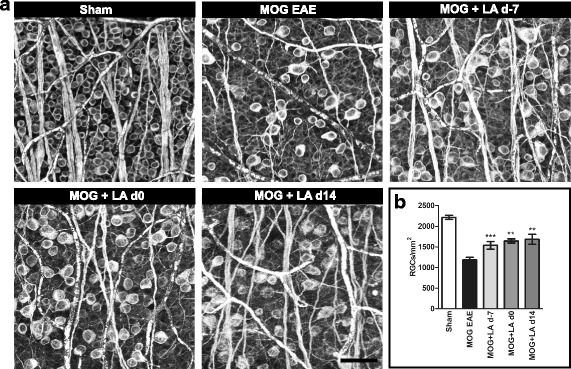
In line with the results of the OCT measurements, LA treatment is beneficial for RGC survival in a MOG EAE in C57BL/6J. One hundred twenty days after immunization with MOG peptide, mice were sacrificed and an enucleation was performed followed by isolation and mounting of the retinae. RGCs were stained with βIII-tubulin antibody (**a**). The bar graph shows the RGC cell number 120 days after immunization; bar = 50 μm (**b**). The bar graph represents the pooled mean ± SEM of three separate EAE experiments each with at least four animals per group; one retina per mouse was included. (*p < 0.05; ***p < 0.001, by ANOVA with Dunnett’s post hoc test compared to MOG-untreated mice)

To elucidate, if the more severe IRL thinning and vision loss in therapeutically treated mice was a direct consequence of the degeneration of retinal ganglion cells (RGCs) or rather of the axons in the RNFL and/or the dendritic arbor in the IPL, we performed histological immunostainings of RGCs in retinal flat mounts using βIII-tubulin (Fig. 5a). The number of RGCs after 120 days was significantly lower (*p* < 0.001, one-way ANOVA analysis) in EAE mice (1181 cells/mm^2^) compared to the sham-EAE control group (2214 cell/mm^2^) confirming the results of the OCT scans. Interestingly, prophylactic (*p* < 0.001, one-way ANOVA analysis) as well as therapeutic LA treatment (*p* < 0.01, one-way ANOVA analysis) led to a higher viability of the RGCs compared to untreated EAE mice (Fig. 5b).

### LA is anti-inflammatory but does not affect demyelination

**Fig. 6:**
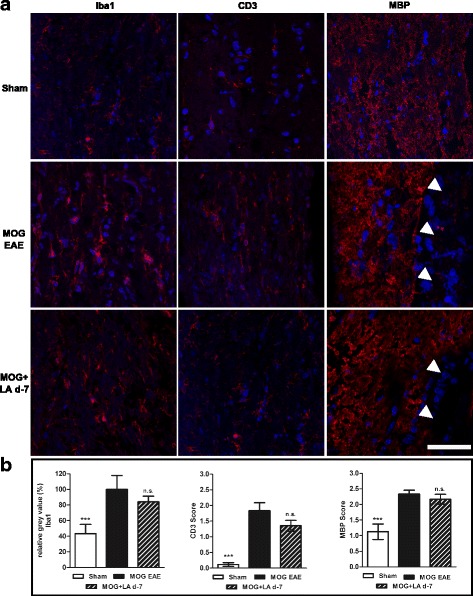
Microglial activation and T cell infiltration were not altered in optic nerves of EAE mice after LA treatment. LA also failed to reduce demyelinating lesions. One hundred twenty days after MOG immunization, mice were sacrificed and optic nerves were extracted and processed, and longitudinal sections were prepared for Iba1, MBP, and CD3 staining (**a**). Optic nerves of sham-EAE, MOG EAE, and MOG EAE with LA-treated mice were compared for microglia activation by fluorescence intensity measurement, by an MBP myelination score for myelin status, and by a CD3 score for T cell Infiltration; quantitative analyses of the results of three independent EAE experiments with at least four mice are shown as bar graphs; bar = 50 μm (**b**); one optic nerve per mouse was included. (***p < 0.001, n.s. = not significant, by ANOVA with Dunnett’s post hoc test compared to MOG-untreated mice)

As LA can reportedly reduce pro-inflammatory cytokines in relapsing-remitting MS [17], we performed histological analyses of immune cell infiltrates in optic nerve sections. We used antibodies directed against Iba1 and CD3, to stain for macrophages/microglia and CD3^+^ lymphocytes, respectively (Fig. 6a). No significant reduction of microglial/macrophage infiltration and activity or infiltration of CD3^+^ T lymphocytes was observed in the mouse optic nerves that had been prophylactically treated with LA as compared to untreated EAE mice. There was, however, a non-significant trend towards a decreased activation/infiltration (Fig. 6b). Therapeutic LA treatment had no effect on microglial/macrophage or lymphocyte infiltration in the optic nerve (see Additional file 4: Figure S3a–b).

To analyze the degree of (de)myelination, we performed immunohistological stainings against the MBP protein. Optic nerves immunized with MOG peptide exhibited large areas of demyelination while sham-EAE mice showed a uniform MBP expression pattern after 120 days (Fig. 6b). The optic nerve myelin status, rated by investigators blinded for the experimental groups, was improved neither after prophylactic (Fig. 6b) nor after therapeutic (see Additional file 4: Figure S3c) LA therapy.

**Fig. 7:**
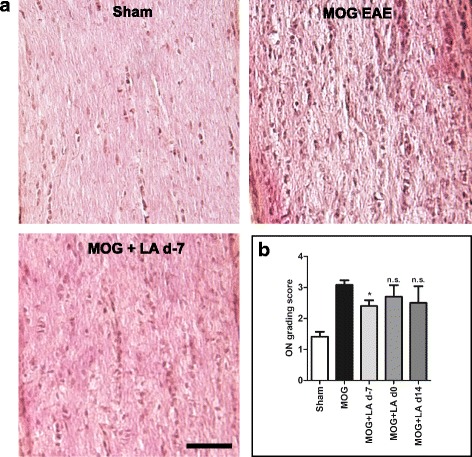
Immune cell infiltration into the optic nerves was reduced after LA treatment. One hundred twenty days after MOG immunization, mice were sacrificed and optic nerves were extracted and processed and longitudinal cuts were prepared for HE staining, bar = 100 μm (**a**). Optic nerves from sham, MOG, and MOG with LA-treated mice were compared by an established infiltration score [41]; results are shown in the bar graph (**b**). The bar graph represents the pooled mean ± standard deviation of at least two separate EAE experiments each with at least four animals per group; one optic nerve per mouse was included (*p < 0.05, n.s. = not significant, by ANOVA with Dunnett’s post hoc test compared to MOG-untreated mice)

HE staining was then performed to investigate the overall infiltration of immune cells into the optic nerves (Fig. 7a). An investigator blinded for the experimental groups rated the degree of severity using an established score [41]. Optic nerves of the sham-immunized control group showed normal histology, whereas MOG immunization resulted in a severe infiltration of inflammatory cells, confirming an established neuritis in 92% of analyzed optic nerves/animals. Pretreatment with LA 7 days before immunization resulted in a significant reduction of infiltrates compared to the optic nerves of the untreated MOG group, while a later LA therapy (d0 or d14) had no effect on the number of immune cells (Fig. 7b).

## Discussion

In multiple sclerosis (MS), permanent disability mainly results from neuronal degeneration which occurs already early on in the course of disease. There is still an unmet need for substances to prevent this degeneration and to prevent permanent disability. As oxidative damage is thought to play an important role in the pathogenesis of neurodegeneration [1], substances with antioxidant properties may be suitable therapeutics. LA is a natural antioxidant available as an oral food supplement. It has an excellent safety profile and has proven to be an effective therapy for EAE [30, 32, 35, 43]. Furthermore, in a recent randomized, placebo-controlled phase II clinical study, brain volume loss over 2 years measured by magnetic resonance imaging was significantly reduced in SPMS under oral treatment with 1200 mg LA per day compared to placebo [18].

The (R)-racemic form of LA is naturally occurring [44] and has been suggested to be superior to the (S)-enantiomer in terms of bioavailability [45–48]. To investigate their neuroprotective capacities independently of a possible immunomodulatory mode of action, we first compared these different forms of LA in a model of endogenous oxidative glutamate stress in HT22 cells. We found that both enantiomers have identical antioxidative effects and that the racemic mixture can be used for further investigations. We then selected 25 μM LA (~ 5.2 μg/mL) for further in vitro investigations as this concentration is achieved in the serum of patients during oral therapy [31]. We determined the dynamics of the protection against oxidative damage by incubating HT22 cells starting from different time points, prior and after glutamate addition. Albeit the (R)-enantiomer of LA is reduced to DHLA 28 times faster by mitochondrial lipoamide dehydrogenase than (S)-LA [49] in our hands, (R)-LA had no superior effect in cell culture. Using DHLA, the neuroprotective capacity was already saturated when the drug was administered simultaneously with glutamate. This is consistent with the assumption that LA has to be reduced to develop its antioxidative potential. However, the difference between the racemic mixture of LA and DHLA was only 24 h, which is most likely irrelevant in a clinical context. Moreover, because racemic LA had a good safety profile in clinical studies, even at very high doses (1200 mg/day), we decided to carry out the in vivo studies with the commercially available racemic form of LA.

Our aim was to investigate if LA is also neuroprotective during the early, inflammatory disease stages of relapsing MS. Phase II and III studies with clinical endpoints related to neuroprotection are extremely time consuming and costly as neuronal degeneration in MS is a slow process and disability progresses over months and years. This deficiency of substitute models for clinical progression has limited the design of neuroprotection trials in MS. Preclinical study designs with readouts directly transferable to clinical trials are therefore of increasing interest. Our experiments aimed to evaluate whether a clinical optic neuritis trial could be reasonable since similar existing preclinical studies [35] lack of visual system readouts.

Moreover, the effect size of an OCT outcome is much higher in a study on optic neuritis where a greater loss of thickness occurs over a much shorter time period [24, 50, 51], therefore largely increasing the chances of a positive result. The MOG peptide-induced EAE-ON model used in the present study constitutes an ideal model to analyze the pathology occurring after an acute ON. It was therefore important to clarify whether the outcome of a previously published study [35] could be copied in our EAE-ON model with visual in vivo readouts as these are broadly used in ON studies. We tested if therapeutic or only prophylactic LA treatment during EAE might have a protective potential. The clinical EAE score was significantly improved after LA treatment, conclusive with many other studies [32, 35, 43], which, however, did not apply in vivo visual readouts. It was therefore crucial to analyze these animals by longitudinal methods such as OCT and OKR [32, 35, 43]. LA treatment starting at the same day or 7 days after MOG injection still had a beneficial effect on the clinical score but was less prominent as compared to early therapy. The inner retinal layers consisting of the retinal nerve fiber layer, the ganglion cell layer, and the inner plexiform layer containing their dendritic arbor is the ideal structure to study neuroprotection and retinal thinning in MS as all these layers show thinning in the context of MS and ON [19–21].

Over a period of 120 days, inner retinal layer degeneration and visual function loss of EAE mice treated with LA prophylactically (d-7) or at the day of immunization (d0) were reduced compared to untreated EAE mice. We found a link between the degeneration of the inner retinal layers and the loss of visual acuity in the EAE mice. A correlation between EAE score and vision loss was also observed in similar studies [52–54]. Single outliners might be due to the occurrence of optic neuritis in the absence of a clinical disability of the animals.

In order to assess the severity of RGC damage at later time points of therapy, we performed βIII-tubulin staining of retinal flat mounts and counted their numbers. Remarkably, but in line with a previous study [35], the RGCs were protected to the same level by a therapeutic and prophylactic LA treatment. Even though the RGCs were little harmed after therapeutic treatment, visual function was not preserved and it had no effect on IRL thinning indicating an irreversible damage occurring at the very early phase of disease and a delayed protective effect of LA. Such discrepancy between the OCT outcome and RGC counts may be explained by a degeneration of the inner plexiform and retinal nerve fiber layers, which cannot be prevented by a treatment starting at the onset of symptoms while the cell bodies of the neurons remain intact. Robust protective effects have also been found by other researchers in a RGC line after hydrostatic pressure [55] and serum deprivation-induced injury, as well as in vivo after an optic nerve crush [56], suggesting a neuroprotective mode of action based on its antioxidant capacities. These abilities are generated by (1) direct scavenging of ROS and free radicals [43]; (2) the induction of detoxification enzymes, like NAD(P)H:quinone oxidoreductase and glutathione-S-transferase [57]; and (3) the increase of the GSH levels in the tissue [58]. In line with this, we observed increased GSH levels under prophylactic LA therapy in the brain of our mice.

However, besides its neuroprotective capacities, the decreased infiltrates observed in our HE-stained sections suggest additional anti-inflammatory effects. Anti-inflammatory properties of LA have so far been attributed to inhibitory effects of LA on T cell migration as it has been reported to inhibit matrix metalloproteinase-9 and the adhesion molecules ICAM-1 and VCAM-1 on the endothelium of the central nervous system [35]. Additionally, it was shown to increase cAMP levels in natural killer cells and thereby inhibiting their function [32]. The fact that in our study numbers on the infiltration of CD3^+^ lymphocytes, on microglia presence, and on degree of demyelination failed to reach significance may be due to the sample size, which was powered for the in vivo readout and on RGC staining.

## Conclusions

Therapeutic treatment with LA attenuates the clinical disability and preserves the survival of RGCs in the EAE-ON model. However, only a prophylactic therapy is capable of preserving visual function and of positively influencing OCT outcomes. We therefore conclude that LA might not be the ideal substance to be investigated in a clinical ON trial, which would use very similar in vivo readouts starting treatment after the onset of clinical symptoms. Nevertheless, our data provide strong evidence that a prophylactic treatment is superior to a therapeutic one and that it is effective already during the first relapse. This supports the concept of an early LA therapy, which should be started as early as possible and could be administered add-on to an immunomodulatory treatment.

## Additional files

Additional file 1: **Table S1.** Advised Protocol for OCT Study Terminology and Elements (APOSTEL) checklist; each item discussed in the manuscript on the indicated page. (PDF 30 kb)

Additional file 2: **Figure S1.** LA treatment days before glutamate induced oxidative stress did not result in improved HT22 protection compared to a 24 h pre-incubation. 5000 cells were seeded into 96-well plates and pre-incubated 9 days (a), 6 days (b) or 2 days (c) before glutamate addition either with vehicle or 25 μM LA DHLA, (R)-LA or (S)-LA. Graphs represent curve fits ±SEM of four independent experiments, each performed in triplicates. Significant differences between vehicle- and substance-treatment are indicated by asterisks (***p < 0.001, area under the curve compared by ANOVA with Dunnett’s post hoc test). (PDF 256 kb)

Additional file 3: **Figure S2.** The carbonylation of proteins did not change 120 days after EAE immunization. The bar graphs represent the pooled mean ± standard deviation of at least three separate EAE experiments each with at least three animals per group (n.s. = not significant, by ANOVA with Dunnett’s post hoc test compared to MOG untreated mice). (PDF 165 kb)

Additional file 4: **Figure S3.** Optic nerves of sham-EAE, MOG-EAE and MOG-EAE with therapeutic LA treated mice were compared for microglia activation by fluorescence intensity measurement (a), by a CD3 score for T-cell Infiltration (b) and by a MBP myelination score for myelin status (c); quantitative analyses of the results of three independent EAE experiments with at least four mice are shown as bar graphs; one ON per mouse was included. (***p < 0.001, n.s. = not significant, by ANOVA with Dunnett’s post hoc test compared to MOG untreated mice). (PDF 227 kb)

## Abbreviations

ANOVA: Analysis of variance
ART: Automatic real time
BW: Bodyweight
c/d: Cycles per degree
CFA: Complete Freund’s adjuvant
DHLA: Dihydrolipoic acid
DMEM: Dulbecco’s modified Eagle medium
DMSO: Dimethylsulfoxid
EAE: Experimental autoimmune encephalomyelitis
EDSS: Expanded disability status scale
ETDRS: Early treatment of diabetic retinopathy study
GCL: Ganglion cell layer
glu: Glutamate
GSH: Glutathione
HE: Hematoxylin and eosin
IPL: Inner plexiform layer
IRL: Inner retinal layer
LA: Alpha-lipoic acid
MBP: Myelin basic protein
MOG_35–55_: Myelin oligodendrocyte glycoprotein fragment 35–55
MS: Multiple sclerosis
NO: Nitric oxide
OCT: Optical coherence tomography
OKR: Optokinetic response
ON: Optic neuritis
PBS: Phosphate-buffered saline
PCBV: Percent change of brain volume
PFA: Paraformaldehyde
PTX: Pertussis toxin
RGCs: Retinal ganglion cells
RNFL: Retinal nerve fiber layer
ROS: Reactive oxygen species
SD-OCT: Spectral-domain OCT
SPMS: Secondary progressive multiple sclerosis
