# Supplementary material for: Early alpha-lipoic acid therapy protects from degeneration of the inner retinal layers and vision loss in an experimental autoimmune encephalomyelitis-optic neuritis model
Source: J Neuroinflammation. 2018 Mar 7;15:71. doi: 10.1186/s12974-018-1111-y (PMC5840773; doi:10.1186/s12974-018-1111-y)
Supplement: Supplementary file 1 — Table S1. Advised Protocol for OCT Study Terminology and Elements (APOSTEL) checklist; each item discussed in the manuscript on the indicated page. (PDF 30 kb) [file 12974_2018_1111_MOESM1_ESM.pdf]

Table S1 Advised Protocol for OCT Study Terminology and Elements (APOSTEL) checklist; each item discussed in the manuscript on the indicated page.

**Item**		**Recommendation**	**Page**
1	Study protocol	Describe how many OCT operating sites and graders were included	1, 13
		Report the timing of OCT compared to other measurements (same day, delayed)	8
		Describe the inclusion and exclusion criteria (here: strain, gender, age)	4
2	Acquisition device	For all OCT devices used, report data on:	
		Manufacturer	4
		Model	4
		Version	4
		Software version	5
3	Acquisition Settings	Clearly describe the settings in which OCT scans were obtained:	
		Room light conditions	4
		Pupils dilated before examination (y/n)	4
		Number of operators and devices	4, 13
4	Scanning protocol	Clearly describe the scanning protocol, including:	
		Type of scan (circular, volume, star, line, other)	5
		Location (area of interest, macula, optic nerve head, papillomacular bundle, other?)	5
		Scan parameters (with or without eye tracking)	5
		Volume scan: size of scan area (degrees or millimeters), number of B-scans, alignment of B-scans, number of A-scans per B-scan	5
		Radial scan: size of scan area (degrees or millimeters), number of B-scans, alignment of B-scans, number of A-scans per B-scan	n. a.
		Ring scan: diameter, A-scans/B-scan, manual or automatic placement of ring or method of centering, depth resolution	n. a.
		Line scan: angle, location, number of A-scans, depth resolution	n. a.
5	Funduscopic imaging	Report other imaging modalities used in addition to OCT (funduscopy, confocal scanning laser ophthalmoscopy, retinal angiography, autofluorescence imaging)	n. a.
		Describe acquisition protocol, including:	
		Excitation wavelength	n. a.
		Filter sets	n. a.
		Number of frames averaged (if applicable)	n. a.
6	Postacquisition data selection	Describe image selection process, including:	
		Quality control criteria (i.e., OSCAR-IB or other criteria)	5
		Postacquisition discard (number and criteria)	n.a.
		Eye selection strategy (if applicable)	n.a.
7	Postacquisition analysis	Describe all postacquisition steps:	
		Software used for processing scans and segmentation (may be different from acquisition software)	5

		Which individual retinal layers were segmented/included	5
		Method of segmentation (automated, semiautomated, or manually)	5
		How potential bias was addressed in the case of manual segmentation (masking)	5
		Grid used for data extraction (size, shape, selected sections)	5
8	Nomenclature and abbreviations	Define:	
		Anatomical structures analyzed	5
		Units of provided measurements (e.g., volume or thickness)	5
9	Statistical approach	Describe:	
		Statistical models used for the analyses of OCT data	7
		Whether data were analyzed by eye or by patient	7

Abbreviation: n. a. = not applicable
