# Supplementary material for: Early alpha-lipoic acid therapy protects from degeneration of the inner retinal layers and vision loss in an experimental autoimmune encephalomyelitis-optic neuritis model
Source: J Neuroinflammation. 2018 Mar 7;15:71. doi: 10.1186/s12974-018-1111-y (PMC5840773; doi:10.1186/s12974-018-1111-y)
Supplement: Supplementary file 2 — Figure S1. LA treatment days before glutamate induced oxidative stress did not result in improved HT22 protection compared to a 24 h pre-incubation. 5000 cells were seeded into 96-well plates and pre-incubated 9 days (a), 6 days (b) or 2 days (c) before glutamate addition either with vehicle or 25 μM LA DHLA, (R)-LA or (S)-LA. Graphs represent curve fits ±SEM of four independent experiments, each performed in triplicates. Significant differences between vehicle- and substance-treatment are indicated by asterisks (***p < 0.001, area under the curve compared by ANOVA with Dunnett’s post hoc test). (PDF 256 kb) [file 12974_2018_1111_MOESM2_ESM.pdf]

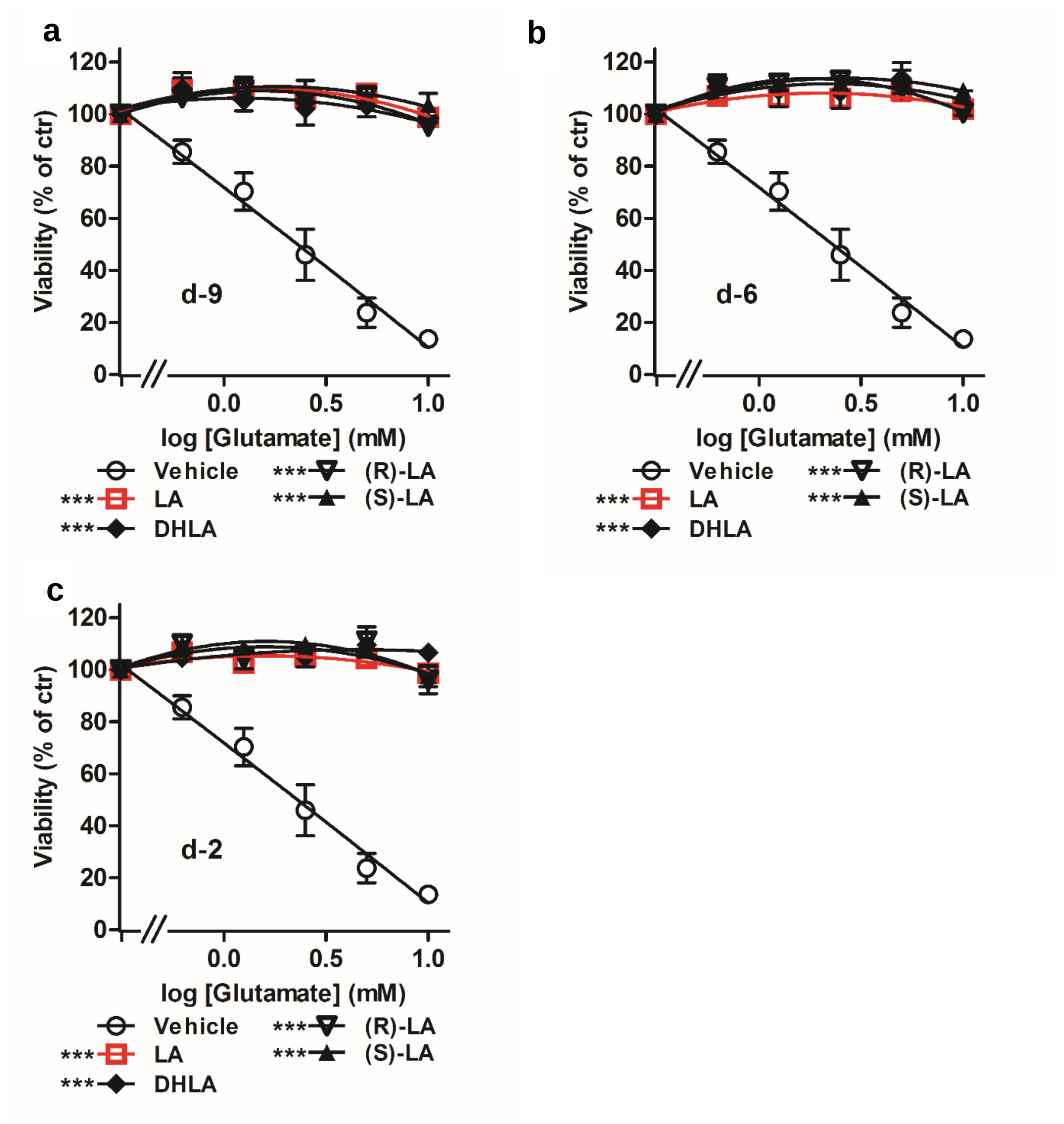

Figure S1 LA treatment days before glutamate induced oxidative stress did not result in improved HT22 protection compared to a 24h pre-incubation. 5000 cells were seeded into 96-well plates and pre-incubated 9 days (a), 6 days (b) or 2 days (c) before glutamate addition either with vehicle or 25 µM LA DHLA, (R)-LA or (S)-LA. Graphs represent curve fits ±SEM of four independent experiments, each performed in triplicates. Significant differences between vehicle- and substance-treatment are indicated by asterisks (***$p<0.001$, area under the curve compared by ANOVA with Dunnett's post hoc test)
