# Supplementary material for: Early alpha-lipoic acid therapy protects from degeneration of the inner retinal layers and vision loss in an experimental autoimmune encephalomyelitis-optic neuritis model
Source: J Neuroinflammation. 2018 Mar 7;15:71. doi: 10.1186/s12974-018-1111-y (PMC5840773; doi:10.1186/s12974-018-1111-y)
Supplement: Supplementary file 3 — Figure S2. The carbonylation of proteins did not change 120 days after EAE immunization. The bar graphs represent the pooled mean ± standard deviation of at least three separate EAE experiments each with at least three animals per group (n.s. = not significant, by ANOVA with Dunnett’s post hoc test compared to MOG untreated mice). (PDF 165 kb) [file 12974_2018_1111_MOESM3_ESM.pdf]

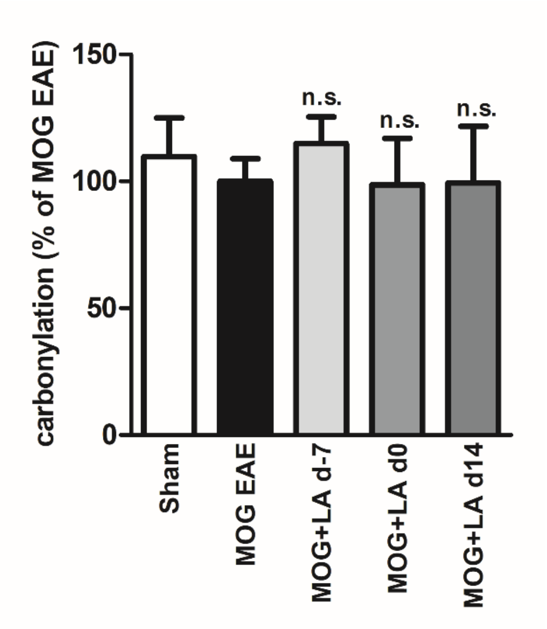

Figure S2 The carbonylation of proteins did not change 120 days after EAE immunization. The bar graphs represent the pooled mean ± standard deviation of at least three separate EAE experiments each with at least three animals per group (n.s. = not significant, by ANOVA with Dunnett's post hoc test compared to MOG untreated mice)
