# Supplementary material for: Early alpha-lipoic acid therapy protects from degeneration of the inner retinal layers and vision loss in an experimental autoimmune encephalomyelitis-optic neuritis model
Source: J Neuroinflammation. 2018 Mar 7;15:71. doi: 10.1186/s12974-018-1111-y (PMC5840773; doi:10.1186/s12974-018-1111-y)
Supplement: Supplementary file 4 — Figure S3. Optic nerves of sham-EAE, MOG-EAE and MOG-EAE with therapeutic LA treated mice were compared for microglia activation by fluorescence intensity measurement (a), by a CD3 score for T-cell Infiltration (b) and by a MBP myelination score for myelin status (c); quantitative analyses of the results of three independent EAE experiments with at least four mice are shown as bar graphs; one ON per mouse was included. (***p < 0.001, n.s. = not significant, by ANOVA with Dunnett’s post hoc test compared to MOG untreated mice). (PDF 227 kb) [file 12974_2018_1111_MOESM4_ESM.pdf]

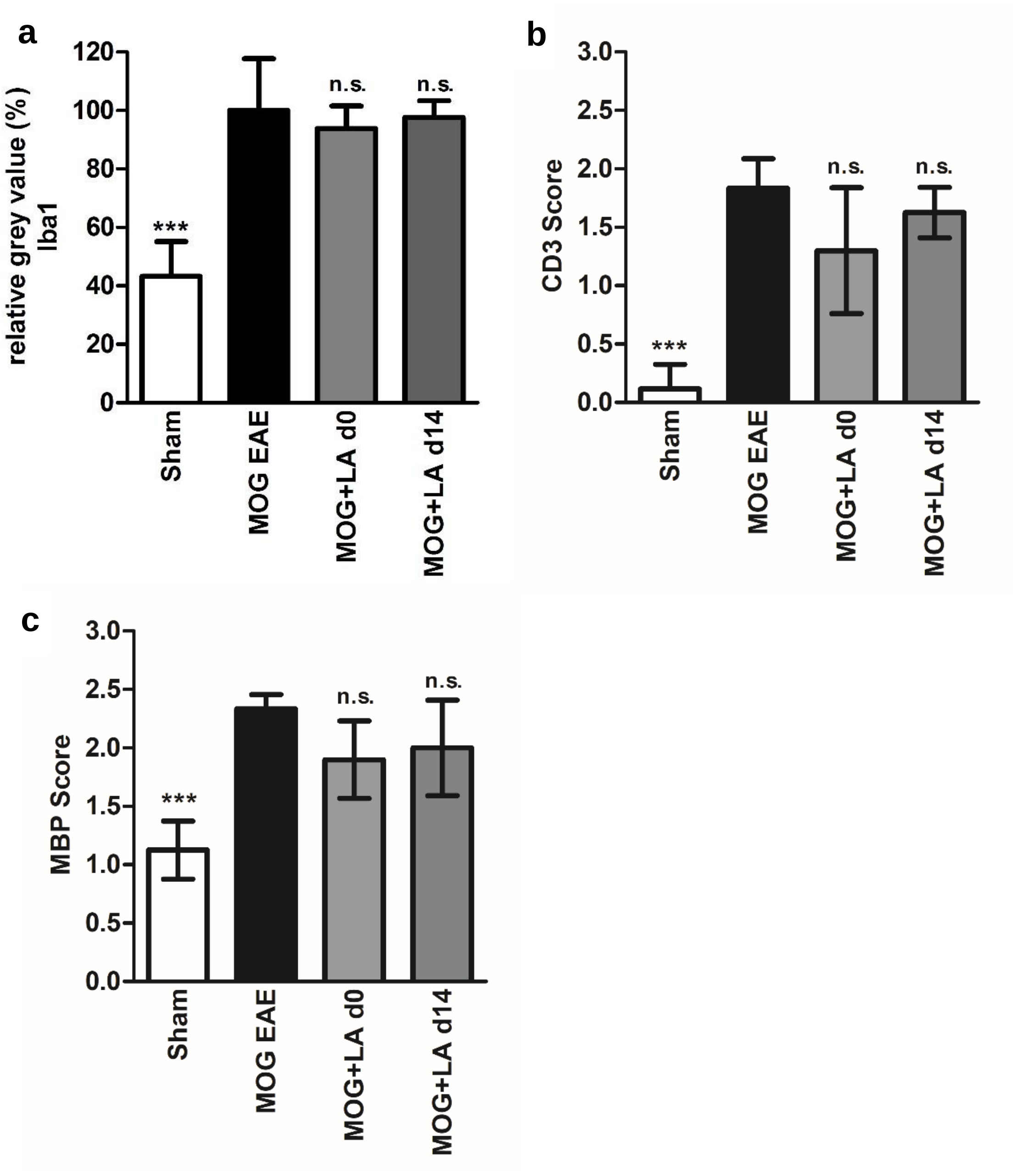


**Figure S3** Optic nerves of sham-EAE, MOG-EAE and MOG-EAE with therapeutic LA treated mice were compared for microglia activation by fluorescence intensity measurement (a), by a CD3 score for T-cell Infiltration (b) and by a MBP myelination score for myelin status (c); quantitative analyses of the results of three independent EAE experiments with at least four mice are shown as bar graphs; one ON per mouse was included. (***p<0.001, n.s. = not significant, by ANOVA with Dunnett's post hoc test compared to MOG untreated mice)
